# Durability Properties of Ultra-High Performance Lightweight Concrete (UHPLC) with Expanded Glass

**DOI:** 10.3390/ma14195817

**Published:** 2021-10-05

**Authors:** Cristin Umbach, Alexander Wetzel, Bernhard Middendorf

**Affiliations:** Institute for Structural Engineering, Department of Structural Materials and Construction Chemistry, University of Kassel, 34127 Kassel, Germany; alexander.wetzel@uni-kassel.de (A.W.); middendorf@uni-kassel.de (B.M.)

**Keywords:** freeze-thaw resistance, acid resistance, abrasion resistance, carbonation, chloride penetration resistance

## Abstract

It is important to ensure the durability and safety of structures. In the case of newly developed materials that are outside the current rules, it is important to investigate all aspects of structural safety. The material studied in the following is a structural lightweight concrete with an ultra-high-performance matrix and expanded glass as a lightweight aggregate. The material, with a compressive strength of 60–100 MPa and a bulk density of 1.5–1.9 kg/dm^3^, showed high capillary porosities of 12 vol% (ultra-high-performance concretes (UHPC) < 5 vol%). Since the capillary porosity basically enables transport processes into the concrete, the material had to be examined more closely from the aspect of durability. Freeze-thaw resistance (68 g/m^2^) and chemical attack with sulfate at pH 3.5 for 12 weeks (16 g/m^2^) showed no increase in concrete corrosion. Targeted carbonation (0.53 mm/year^0.5^) and chloride penetration resistance (6.0 × 10^−13^ to 12.6 × 10^−13^ m^2^/s) also showed good results against reinforcement corrosion. The results show that most of the measured capillary pores resulted from the lightweight aggregate and were not all present as a pore system. Thus, the durability was only slightly affected and the concrete can be compared to an UHPC. Only the abrasion resistance showed an increased value (22,000 mm^3^/5000 mm^2^), which, however, only matters if the material is used as a screed.

## 1. Introduction

High-strength (HPC) and ultra-high-performance concretes (UHPC) offer the possibility to build higher, more filigree, and in larger distances between piles or other supporting structures [1]. Further weight savings through the use of ultra-high-performance lightweight concrete (UHPLC) opens up new architectural possibilities. Additionally, in repair work where the weight of the overall structure is a key factor in order to avoid expensive and usually long-lasting foundation work, the use of lightweight concrete can be more economical. Lightweight construction materials can also facilitate the transport and installation of massive new buildings made of precast elements, such as tribunes of stadiums or bridges. The disadvantage of lightweight concretes is their comparatively lower mechanical performance and higher cost [2]. For this purpose, the concepts of ultra-high-performance concrete and lightweight concrete were combined.

UHPC is a high-performance building material characterized by a compressive strength of more than 150 MPa and a very dense microstructure [3]. Due to its very high strength, UHPC is not defined by the standard EN 206 [4]. In Europe, some countries, such as France [5,6] and Switzerland [7], have already established regulations for the use of UHPC. In Germany, there is still no valid standard for this material [3,8,9,10]. However, the properties of UHPC have been investigated in research programs. One of the most extensive was the research carried out by German Research Foundation (DFG) in the German Priority Program SPP 1182, which is summarized in [1]. The development of the material investigated in the following is based on the fine-grain UHPC mix M3Q used in this study.

To design a UHPC, the following requirements have to be fulfilled: a low water/binder ratio (<0.25), optimized packing of the raw materials, and reactive additives. The use of high-performance superplasticizers based on polycarboxylate ethers (PCE) is necessary to make the material processable with these requirements. Faust describes in [2] that a high-performance or ultra-high-performance matrix can increase the strength of lightweight concrete.

In addition to the mechanical properties, the durability properties are also essential for the safety of structures. This is especially important for materials that are outside the current regulations and standards. Here it is necessary to ensure that the durability is achieved according to the exposure of the material. Therefore, the material is investigated in the following with regard to its resistance to concrete and reinforcement corrosion.

A durable concrete is characterized by its resistance to chemical or physical attack without suffering any damage. Damage caused by these attacks has a significant impact on the safety and serviceability of the structure as well as its appearance. In addition to mechanical stress, it is therefore important to prevent the penetration of fluids into the concrete. Excess water, which is not incorporated into hydration phases, creates capillary pores. Along these capillary pores, transport processes into the concrete are enabled. Basically, the damage caused by insufficient durability can be divided into two categories: reinforcement corrosion and concrete corrosion. In standard concrete, according to EN 206 [4], fluids can penetrate the concrete via the capillary pores, the contact zone between aggregate and hardened cement paste, and microcracks. For this reason, the standard demands not only a minimum compressive strength but also a maximum water/cement ratio (w/c ratio) in order to reduce capillary porosity and maintain durability.

Lightweight aggregates are used in concrete to reduce the bulk density to below 2.0 kg/dm^3^ to obtain a lightweight concrete. This affects the mechanical performance of the concrete, as the load transfer is disturbed by the porous structures. In addition, the type of lightweight aggregate influences the fresh and hardened concrete properties. Expanded clay is a very frequently used lightweight aggregate. The porous grains have a closed sintered skin and a relatively high strength of 3–8 MPa, but also a high grain density of 0.8–1.8 kg/dm^3^. A disadvantage is the large grain size of 2–16 mm compared to fine-grained UHPC, which usually has a maximum grain size of 0.5 mm. The available smaller grain sizes result from larger broken fractions. Thus, the water uptake is increased, resulting in a decrease in workability. An alternative lightweight aggregate is perlite, which is available as very fine graded grain fractions, too. The very light material has a grain density of 0.09 kg/dm^3^ and a strength of only 0.2 MPa. Due to a missing sinter skin, these aggregates adsorb a lot of water, which makes it difficult to process at low w/b ratios. Expanded glass has fine graded grain fractions and a sintered skin. Another industrially produced material is expanded glass, which is manufactured in very fine particle sizes. However, the strength of 2.8 MPa (grain size of 0.1–0.3 mm) is rather low.

Structural lightweight concretes are a combination of dense cement paste matrix and lightweight aggregate [4]. Some lightweight aggregates have a high permeability, which at first sight affects the durability [2,4]. However, the interfacial transition zone (ITZ) between the lightweight aggregate and the cement paste matrix is usually denser, as the rough surface of the aggregates creates an improved bond [2,11,12]. The mechanical properties of structural lightweight concrete do not correlate with the w/c ratio, as is the case with standard concrete. Therefore, only the w/c ratio and not the strength is significant for lightweight concretes for the classification in terms of durability according to EN 206 [4].

The material considered in the following is structural lightweight concrete improved with a UHPC structure to generate a high-performance and at the same time lighter construction material. UHPC (M3Q) [1] is a well-studied material whose durability is ensured due to its high structural density. The influence of lightweight aggregate in UHPC on the mechanical as well as the durability properties has not yet been sufficiently investigated, as this is a new class of material.

## 2. Materials

**Figure 1:**
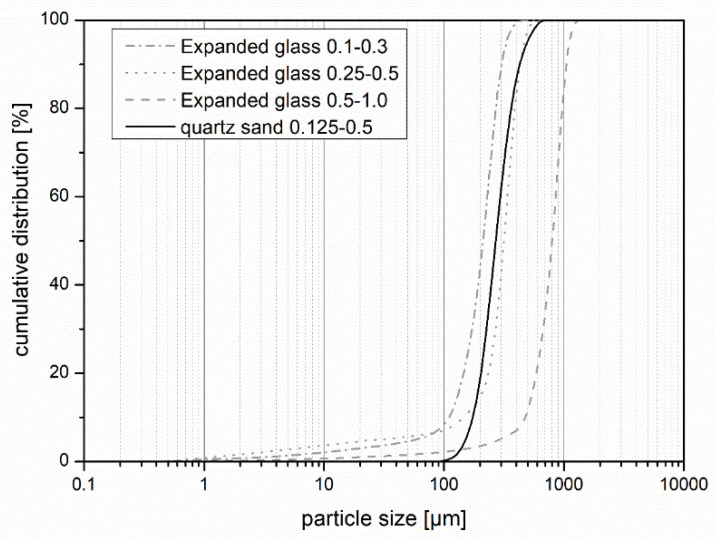
Cumulative particle size distributions of the expanded glass and quartz sand.

In material development, the grain size distribution of the raw materials is important for the mix design of high-performance concretes. Since an existing formulation was modified by the substitution of fines by new raw materials, the size distributions of these new materials were determined and compared to the quartz sand (Figure 1). Expanded glass, which is available in very fine particle sizes, was suitable for replacing the fine aggregate in UHPC (Table 1) The particle size distribution was measured with a laser granulometer LS 13 320 XR from Beckman Coulter GmbH (Brea, CA, USA) using the wet mode. The results were evaluated according to the Fraunhofer theory.

**Figure 2:**
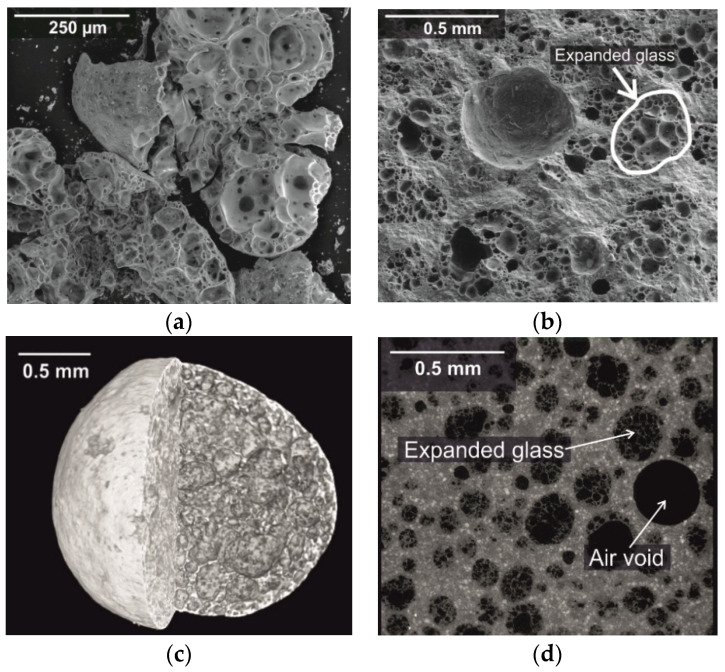
SEM images (SE mode; low vacuum, 50 Pa, 15 kV): (**a**) fractured expanded glass grain and (**b**) expanded glass embedded in UHPC on a fractured surface. CT images: (**c**) surface and internal structure of expanded glass, spatial resolution 1 µm, and (**d**) sectional view of the material structure, spatial resolution 1.5 µm.

It was noticeable that the expanded glass had a larger number of undersize grains. In case of the product with a grain size of 0.25–0.5 mm, this even exceeded 22 vol%. This can be explained by the low grain compressive strength, since the grains broke very easily. Figure 2a,b shows a single broken expanded glass grain (left) and a fractured surface of a concrete containing expanded glass in the hardened cement paste matrix in a scanning electron microscope (Quanta FEG 250 from FEI, Hillsboro, OR, USA). The secondary electron images were acquired in low vacuum and show, on the one hand, the fragility of the expanded glass and the embedding of the whole unbroken grains in the cement paste matrix.

The computed tomography images (Figure 2c) show an exfoliated glass grain as a reconstructed 3D model. Here, the surface structure as well as the porous structure inside can be seen. The second image shows a sectional view of a shot of a concrete mix with expanded glass. Here, too, the good embedding of the expanded glass grains can be seen. Images were taken using a Zeiss Xradia Versa 520 high-resolution computed tomography (µ-CT) scanner form Zeiss Microscopy (Oberkochen, Germany). Data were analyzed using the Avizo 9.5 program from FEI Inc.

Three formulations were considered that differed in the bulk density class to be achieved. The density classes are described, for example, with D1.6, which describes the design raw density of 1.6 kg/dm^3^. The dry density of the material was in this case below 1.6 kg/dm^3^ but above 1.4 kg/dm^3^. The different bulk density classes were achieved by different grain fractions and proportions of expanded glass (EG) in the formulations. The composition of the fine-grained fraction and additives—cement, silica fume, quartz flour, superplasticizer, and hydrophobing agent—remained the same for all formulations (Table 1).

The concrete was mixed in intensive mixers from Eirich (Herdheim, Germany). The dry materials were homogenized, including the expanded glass, for 60 s. Then water, superplasticizer, and hydrophobing agent were added and mixed for 2 min at high intensity (tool speed 3.6 m/s, bin speed 1.0 m/s). In a subsequent homogenization phase, mixing was continued for another 5 min at low intensity (tool speed 1.8 m/s, bin speed 0.5 m/s). After mixing, the concrete was cased into forms and compacted (50 Hz, 120 s, outside vibration compaction). After one day, the specimens were demolded and stored at 20 °C and 65% r.h.

## 3. Methods

### 3.1. Compressive Stength and Bulk Density

The compressive strength was tested according to EN 12390-3 [13]. The samples were cubes with a dimension of 100 mm. The tests were load controlled with a speed of 0.5 N/mm^2^s. The average of three samples was taken. With the determination of the compressive strength, the hardened concrete density was also determined by weighing and measuring the specimens.

### 3.2. Microstructure

Mercury intrusion porosimetry (MIP) is a common method for determining the micro porosity of building materials. With the results, statements can be made about the pore size distribution as well as the pore content. The investigations were carried out in accordance with ISO 15901-1 [14] with a Poremaster from Quantachrome (Boynton Beach, Fl, USA). Prior to measurement, the 28-day-old samples were dried at 40 °C for 24 h and measured in fragments up to 5 mm.

The water impermeability was tested according to EN 12390-8 [15]. For the test, three cubes with a dimension of 150 mm were subjected with the formwork surface to a water pressure of 5 bar for 72 h. After three days, the specimens were split and the penetrated water level was measured.

A Zeiss SV 11 stereomicroscope was used to examine the surfaces in more detail. For this purpose, specimens were viewed in reflected light microscopy.

### 3.3. Resistance to Concrete Corrosion

The concrete corrosion was tested in the following for the case of frost, chemical attack, and abrasion. For all three types of corrosion, a loss of mass was considered, which was expressed in different parameters. To make the results comparable, the corrosion was converted to g/m^2^ for all tests. The freeze-thaw resistance with de-icing salt was determined according to CEN/TS 12390-9 [16] using the capillary suction of de-icing solution and freeze-thaw test (CDF test) method. For this purpose, five specimens with a sawed examination area of 150 × 112 mm^2^ were presaturated in 3% NaCl solution for 7 days prior to testing and then subjected to 28 freeze-thaw cycles in the same test solution. The CDF-CIF-Freeze-Thaw-Tester manufactured by Schleibinger Testing Systems was used. For one freeze-thaw cycle, the samples were frozen at −20 °C and then rewarmed to 20 °C. After 4, 6, 14, and 28 cycles, the weathered material was weighed. The mass loss related to the surface area indicated the freeze-thaw resistance. The total mass loss should be less than 1.5 kg/m^2^. This criterion applies to a normal concrete with a density of 2.1 kg/dm^3^ and should be reduced for lightweight concretes [2]. For the concrete mix EG D1.6, the limit value was therefore 1.5 kg/m^2^ × 1.51/2.1 = 1.08 kg/m^2^.

To investigate the acid resistance of the four samples, they were stored in sulfuric acid at pH 3.5 for 12 weeks according to [17]. Prior to testing, the samples were sawed to a dimension of 150 × 100 × 40 mm^3^ and dry samples were weighed and measured. After that, the samples were presaturated in water for three days. The pH value of the solution was kept constant by titration. The mass of the samples was determined once a week. Loose particles were removed from two of the four samples with a brass brush. The result was the mass loss in % and g/m^2^.

The abrasion resistance was determined according to EN 1339 [18] with grinding wheel N1001 from From+Test (Riedlingen, Germany). The three concrete specimens with a dimension of 71 × 71 × 71 mm^3^ were loaded with 294 N and passed over the abrasive artificial corundum in 4 rounds. One round included 88 rotations of the grinding wheel. After each round, the weight of the remaining specimen was measured and new abrasive was applied to the wheel. After the test, the remaining sample height was also measured. The test result was expressed in mm^3^/5000 mm^2^.

### 3.4. Resistance to Reinforcement Corrosion

The resistance to reinforcement corrosion was initially given in the alkaline environment of the hardened cement paste. The diffusion of gases and chlorides can disrupt this system and reinforcement corrosion can take place.

The carbonation of the UHPLC was determined in accordance with the Swiss standard SIA 262-1, Appendix I [19]. The procedure describes rapid carbonation by a chamber that is selectively filled with CO_2_. The standard specifies a saturation of 4 vol% CO_2_ at 20 °C and 65% r.h. The normal ambient conditions of the outside air were assumed to be 0.4 vol% CO_2_. In addition to a zero measurement (0 d), samples were kept in the rapid carbonation chamber for 7, 28, and 63 days. One day in the chamber corresponded to 100 days of aging in the outside air. This simulated an aging of approx. 2 years in 7 days. After each storage period, fresh fractured surfaces were sprayed with the indicator solution (0.1% phenolphthalein solution) to determine the carbonation depth *d_km_*. Based on this, the coefficient of carbonation *K_N_* was calculated according to Formulas (1) and (2) [19].

(1) $$K_{N}=a\times b\times c\times K_{s}$$

(2) $$K_{S}=\frac{d_{KM}}{\sqrt{t}}$$

*K_N_*Carbonation coefficient under natural conditions (mm/year^−1^)*a*Conversion factor from 1 day to 1 year = $\sqrt{365/1}$ = 19.10*b*Conversion factor from 4.0 auf 0.4 vol% CO_2_ =
$\sqrt{0.04/4.0}=0.10$
*c*Correction factor for raoid carbonation = 1.36*K_S_*Carbonation coefficient at 4.0 vol% CO_2_ (mm/day^−1^)*d_KM_*Mean value of carbonation depth (mm)*t*Time (days/years)

The determination of the chloride penetration resistance was carried out according to the Rapid Chloride Migration test (RCM), which was specified in a leaflet of the Federal Waterways Engineering and Research Institute [20]. The experiment determines the penetration of chlorides in cementitious materials under the influence of an electric field. For this purpose, 50 mm-high specimens were sawn from the center of a cylinder (Ø100, h = 200 mm). The penetration of chloride (test solution: 10% NaCl solution) into three samples under the influence of an electric field with a voltage of 30 V was investigated. The samples were stored for four days in this test setup. At the end of the experiment, the samples were split and sprayed with the indicator solution (0.1% fluorescein solution) and after surface drying, silver nitrate solution was sprayed on. The chloride front thus became visible after a few hours in daylight or UV light. From the results of the experiment, the chloride migration coefficient *D_RCM_* was calculated according to Formulas (3) and (4) [20].

(3) $$D_{RCM}=\frac{R\times T}{z\times F\times E}\times \frac{x_{d}-\alpha \times \sqrt{x_{d}}}{t}$$

(4) $$\alpha =2\times \sqrt{\frac{R\times T}{Z\times F\times E}}\times erf^{-1}\left(1-\frac{2\times c_{d}}{c_{0}}\right)$$

*R*Gas constant, *R* = 8.315 (J/(K-mol))*T*Absolute mean temperature of the solutions during the experiment (K)*z*Ion load, for chloride ions *z* = 1 (-)*F*Faraday’s constant, *F* = 9.649–104 (J/(V-mol))*E*electric field strength (V/m)*x_d_*Mean penetration depth of the chloride ions of the two halves of the specimen (m)*t*Test duration (duration of voltage application) (s)*erf*^−1^Inverse error function*c_d_*Color change-inducing chloride concentration, *c_d_* = 0.07 (mol/L)*c*_0_Chloride concentration of the potassium hydroxide solution (mol/L)

For the determination of the chloride content in concrete, concrete milled to powder was photometrically analyzed. Instructions for this method have been published in [21] by the German Committee for Reinforced Concrete. To dissolve the salt from the powder, 20 g of deionized water and 4 g of nitric acid are added. The resulting solution is dissolved in silver nitrate solution and the resulting coloration is measured. From this, the chloride content can be determined. In this method, the chloride content is determined in wt% in relation to the cement content. According to DIN 1045-2 [22], the limit values for the chloride content are given as 0.4 wt% for reinforced concrete. Prestressed concrete has a stricter limit value of 0.2 wt%.

## 4. Results

### 4.1. Compressive Strength

**Figure 3:**
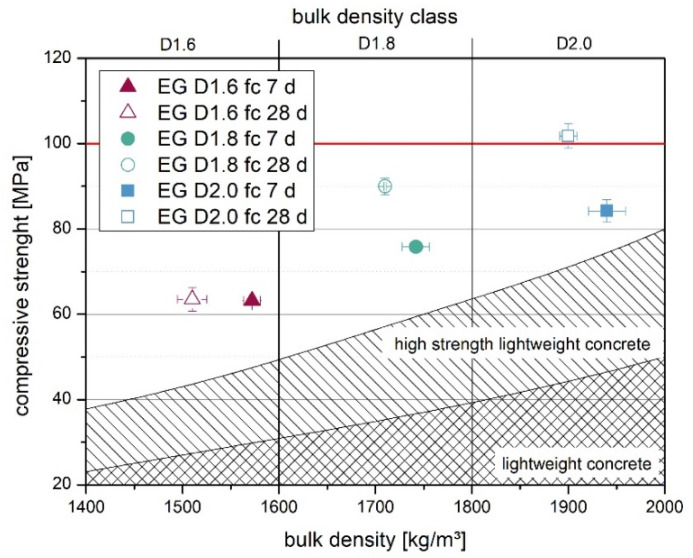
Compressive strength of UHPLC with expanded glass with different bulk density classes. The standard area for lightweight concrete was designed according to Thienel [23].

For lightweight concrete, usually the following trend can be observed: the higher the bulk density, the higher the strength of the lightweight concrete (Figure 3). For the material with a bulk density class of D2.0, the highest strengths of about 100 MPa were obtained after 28 days. As expected, the strength of the bulk density class D1.6 was lower at 63 MPa. Moreover, this concrete reached its final strength after seven days. The final strength of the UHPLC was 100–140 MPa less than the UHPC mix described in [1] achieved. However, this concrete also had a higher bulk density of 2300 kg/m^3^.

### 4.2. Microstructure

**Figure 4:**
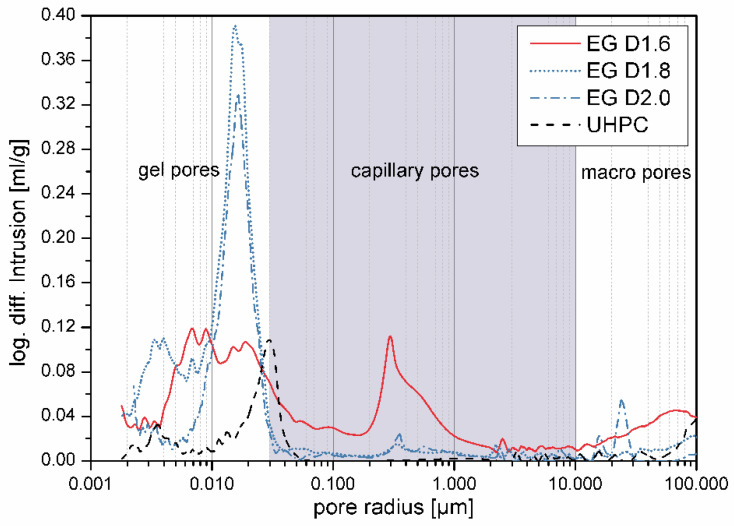
Porosity distribution of UHPC [3] and UHPLC determined by using mercury intrusion porosimetry. Pore size limits according to Setzer [25].

Mercury intrusion porosimetry can be used to compare pore structure below 100 µm for different concretes (Figure 4 and Table 2). In contrast to UHPC [3], UHPLC EG D1.6 contained a high capillary pore peak at ca. 0.3 µm. The total capillary pore content of the material was 12 vol%, which is also much higher than usual in UHPC mixes (<5%). In contrast, the materials EG D1.8 and EG D2.0 showed a high peak at about 0.016 µm in the gel pore area. This peak was also not found in the UHPC reference. The capillary porosity of the two materials was comparable to that of UHPC at around 3%. The peak at 0.3 µm was much lower for these specimens.

Due to the high capillary porosity of the EG D1.6 sample, the water impermeability was tested. The water impermeability test is recommended for white tubs in order to avoid an additional sealing layer in concretes with pressing groundwater [24]. On average, the water penetrated 2–3 mm into the concrete.

The following material tests were carried out on specimens of the EG D1.6 mixture (Table 1) and at a concrete age of 28 days, as the greatest reductions were expected here due to the high capillary porosity.

### 4.3. Freeze-Thaw, Acid, and Abrasion Resistance

**Figure 5:**
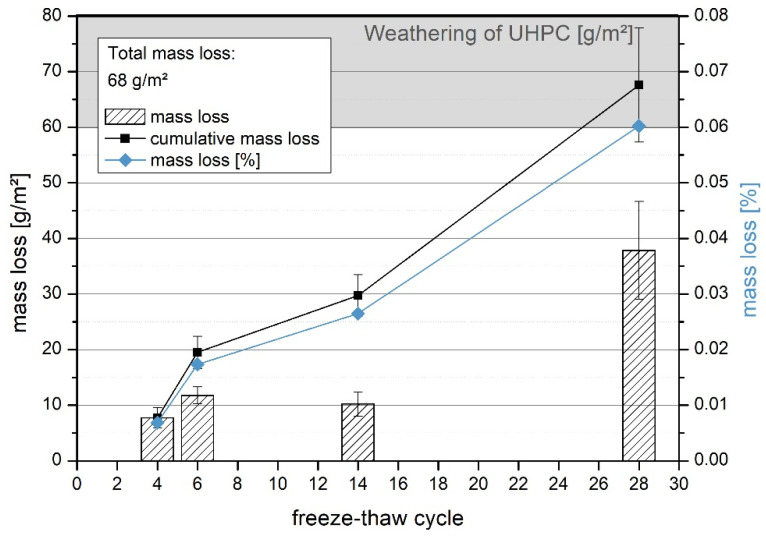
UHPLC EG D1.6 in the freeze-thaw test with de-icing salt. Highlighted in gray is the range of UHPC tests in the literature after 28 cycles [1,26].

In the freeze-thaw tests, the UHPLC specimens showed a mass loss of 1.14 g after 28 cycles (Figure 5). This resulted in a total mass loss of 68 g/m^2^. As a result, the specimens passed this test, according to standard CEN/TS 12390-9 [16]. After 14 cycles, the average weathering was about 0.5 g. Thus, a uniform material behavior was observed over the entire test run.

**Figure 6:**
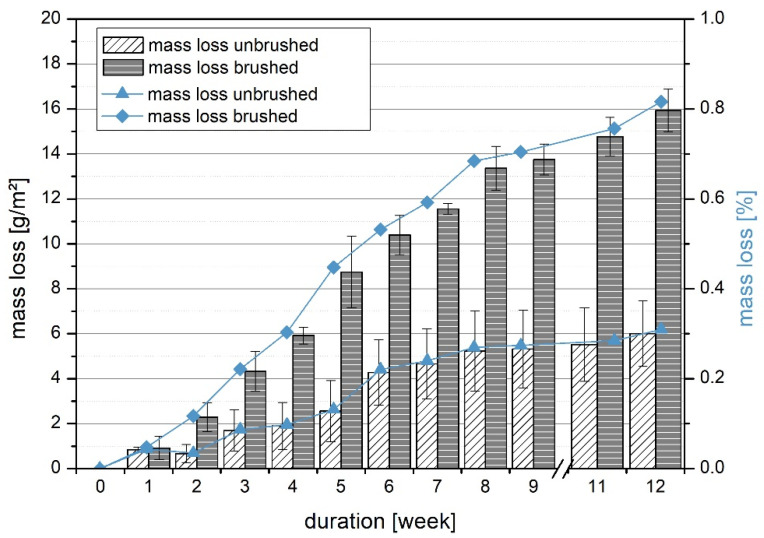
Chemical resistance results of UHPLC EG D1.6 at pH 3.5.

The UHPLC samples also lost mass slowly and continuously in acid storage (Figure 6). The total mass loss was 3.0 g for the unbrushed and 8.1 g for the brushed. This corresponded to wears of 6 g/m^2^ and 16 g/m^2^, respectively. Due to brushing, the aberration was higher and after 12 weeks there was a mass loss of 0.82%.

**Figure 7:**
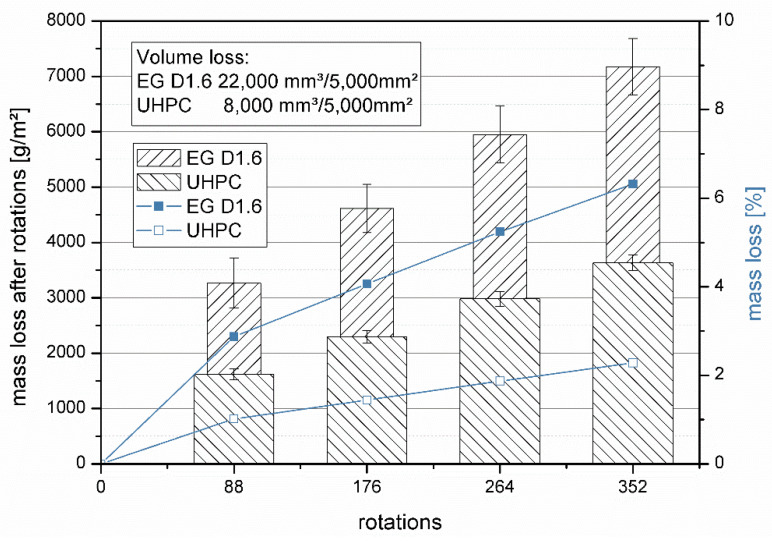
Abrasion test of UHPLC EG D1.6 and UHPC.

The mass loss in the abrasive test of the UHPLC samples was 7175 g/m^2^ (Figure 7). This corresponded to a mass loss of 6.32%. The material also lost 4.5 mm in height. The volume loss was thus 22,000 mm^3^/5000 mm^2^. The UHPC tested in the same way lost 3631 g/m^3^. This corresponded to a mass loss of 2.3% and a volume loss of 8000 mm^3^/5000 mm^2^. Both materials showed similar abrasion.

### 4.4. Carbonation and Chloride Diffusion

**Figure 8:**
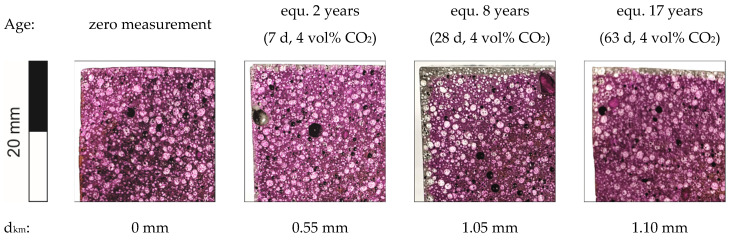
Carbonation of UHPLC EG D1.6 determined in a rapid carbonation chamber after 7, 28, and 63 days.

The determination of the carbonation depth after storage in the rapid carbonation chamber was done at four positions, one on each side of the fracture surface of the cube, and an average value is given in Figure 8.

According to SIA 262-1 Appendix I [19], the carbonation coefficient is determined from the carbonation depth by using rapid carbonation KS. The coefficient was 0.19 mm/day^0.5^. Furthermore, this coefficient can be converted into the carbonation coefficient under natural conditions *K_N_*. For this purpose, three factors were taken into account: the conversion from day to year, the conversion from 4 vol% to 0.04 vol% CO_2_, and a correction factor for the rapid carbonation process. This resulted in *K_N_* being 0.53 mm/year^0.5^. Following this, a carbonation depth *d_km_* of 5.3 mm was calculated for a time period of 100 years.

**Figure 9:**
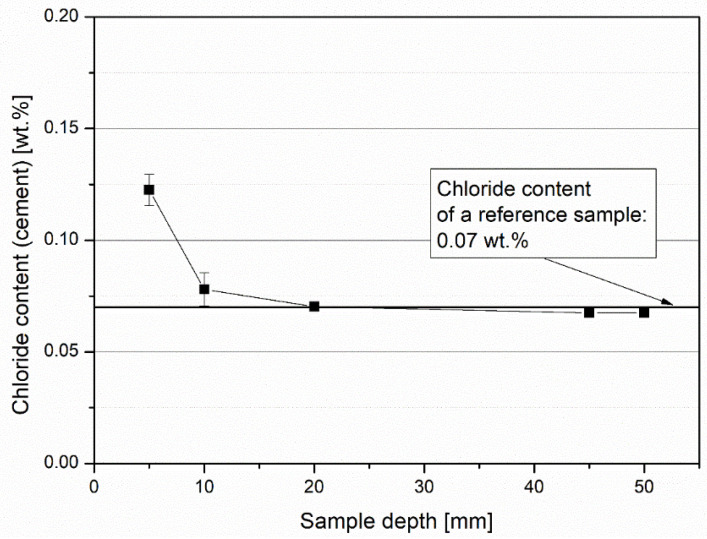
Chloride content based on cement content determined on samples analyzed photometrically after the Rapid Chloride Migration test.

In the Rapid Chloride Migration test, no discoloration was observed when determining the depth of chloride penetration into the concrete after application of the indicator solutions. In order to determine a penetration depth, flour samples were taken at different sample depths. The drill dust was taken at 5, 10, and 20 mm depth on the side exposed to chloride and from the opposite side. On these samples the chloride content was determined photometrically (Figure 9). Due to the very high cement content (772 kg/m^3^), a maximum chloride content of 0.12 wt% was measured. This was below the limit value of 0.5 wt%. Based on the results, a chloride level of 10–20 mm was concluded. This resulted in a chloride diffusion coefficient of 6.0 × 10^−13^ to 12.6 × 10^−13^ m^2^/s.

## 5. Discussion

To classify the tests, the concrete was compared to UHPC and to other lightweight concretes. The material showed high resistance to freeze-thaw attack, as the total mass loss after 28 freeze-thaw cycles of 0.064 kg/m^2^ (Figure 5) was far below the limit of 1.08 kg/m^2^. The material also met the stricter requirement for concrete slabs according to EN 1339 [18] of 0.72 kg/m^3^ (1.0 kg/m^2^ for standard concrete). This means that the material can also be used outdoors in areas with de-icing agents without any further precautions. In [26], Müller et al. describe an overall weathering of UHPC after 28 freeze-thaw cycles with de-icing salt of 60–80 g/m^2^. This means that lightweight concrete has the same frost resistance as UHPC.

The frost resistance of lightweight concretes depends, like any concrete, on the matrix porosity. Therefore, the water/binder value is also essential for frost resistance. Another important factor is the type of lightweight aggregate [2]. In crushed aggregate or in aggregate with a porous surface, the water that is transported into the structure via the capillary pores can penetrate the lightweight aggregate. When freezing, the water can expand here similar to air voids and cause less damage [2,27,28]. However, Lotfy et al. [29] showed that this can also have a negative effect. Here, lightweight concretes with different w/b ratios and expanded clay were tested for frost resistance with de-icing salt. In general, frost resistance decreased with increasing w/b ratios. However, the concretes with expanded clay and high water contents soaked up so much water that they did not pass the frost test because they broke apart.

Against an attack with sulfuric acid at pH 3.5, the material also demonstrated very good resistance, with a maximum of 0.82% mass loss after 12 weeks (Figure 6). Other types of chemical attack must be tested depending on the application. In the case of a chemical attack, it may be advisable to provide a sacrificial concrete layer or coat the concrete, depending on the application [30].

In Müller et al. [26], UHPC was stored at pH 3 for 24 weeks. After this time, a corrosion of 0.79 mm was measured. The corrosion of samples from UHPLC was 0.14 mm. All specimens showed uniform corrosion on the surface. Cracks or other defects such as spalling were not observed. The shorter storage time and the slightly lower pH suggest that the acid resistance was similar to that of UHPC.

In studies on lightweight concretes with different w/b ratios from 0.28 to 0.40, Wang [31] found that chemical resistance to sulfates increased with decreasing w/b ratio, and damages like cracking and corrosion decreased. The weight loss was reduced from 18% to 7%. This shows that the w/b ratio is an important parameter for the chemical resistance and that the type of lightweight aggregate has only a minor effect.

**Figure 10:**
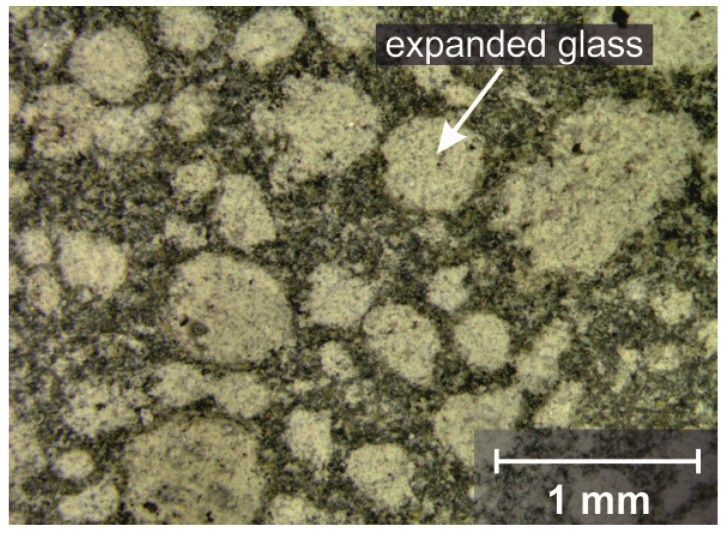
UHPLC under a reflected light microscope after the abrasion test.

Abrasion resistance according to EN 1339 [18] showed a high abrasion of 22,000 mm^3^/5000 mm^2^ (Figure 7). This value can be grouped into different concrete standards, depending on the application. According to EN 1339 [18], the material is to be classified as a concrete slab in class 2 (≤26,000 mm^3^/5000 mm^2^). As a screed, the concrete would be assigned to class A22 according to EN 13813 [32]. This is the highest abrasion class for screeds. UHPC with the same raw materials (without lightweight aggregate) has an abrasion of 8000 mm^3^/5000 mm^2^. Therefore, the wear of 22,000 mm^2^/5000 mm^3^ was due to the expanded glass. Here, the negative influence of the low compressive strength of the lightweight aggregate becomes apparent. The expanded glass abraded quickly and thus the rest of the matrix also abraded more quickly. Under the microscope, it can be seen that a uniform abrasion took place and the expanded glass remained firmly bonded (Figure 10). Therefore, use as a screed for utility areas is not recommended.

The tests in the rapid carbonation chamber (Figure 8) showed a very low coefficient of 0.5 mm/year^0.5^. According to German guidelines, there are no limit values for the carbonation coefficient for concrete. According to SIA 262-1 [19], the strictest requirement is ≤4.0 mm/year^0.5^, which shows that the carbonation progress of this material exceeds the requirement. After 100 years, a carbonation depth of 0.53 mm can be expected. Therefore, a reduction in the concrete cover in case that reinforcement is used could be considered for economic reasons like material saving.

The chloride diffusion coefficient of 6.0 × 10^−13^ to 12.6 × 10^−13^ m^2^/s was also low and would allow structures to come into contact with salt water. This applies to structures in the tidal or spray water area of seawater as well as structures that come into contact with de-icing salt. In the case of traffic roads and structures, however, appropriate protection against abrasion must be ensured.

Compared to UHPC (6 × 10^−14^ to 10 × 10^−14^ m^2^/s [15]) the chloride diffusion coefficient of 6.0 × 10^−13^ to 12.6 × 10^−13^ m^2^/s increased. However, it was significantly lower than normal concrete at 2^−12^ to 25 × 10^−12^ m^2^/s [17]. In [33], coefficients of around 1 × 10^−12^ to 9 × 10^−12^ m^2^/s are described for structural lightweight concretes. Here, the coefficient decreased with increasing silica fume content. The effect can be explained by the improved microstructure. In the case of the UHPLC, the cement paste matrix C creates a dense microstructure, which can prevent chloride diffusion.

**Figure 11:**
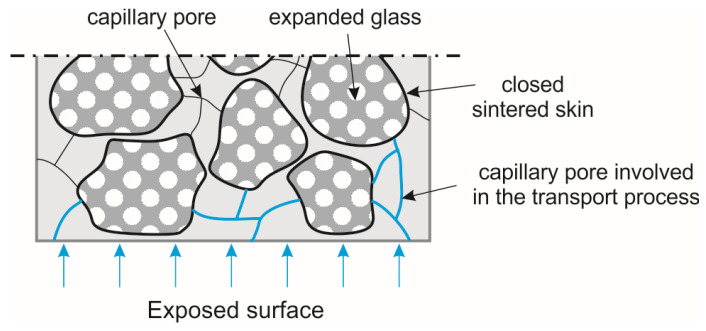
Schematic transport mechanism in UHPLC for the presented mixes.

The durability properties of the investigated material suggest that the high capillary porosity of 12 vol% in the MIP test was not due to structural pores. Instead, the measurement was influenced by the expanded glass. It is assumed that the pores of the lightweight aggregates were measured and caused the peak. The same applies to the peaks in the gel pore area for the mixtures EG D1.8 and EG D2.0. Here, the coarser grain size (0.5/1 mm) was not used and a finer one (0.1/0.3 mm) was used instead. The very low value of about 2 mm in the water impermeability test for the mixture EG D1.6 supports the assumption that the pores around 0.3 µm originated from the expanded glass and were therefore closed. The closed sintered skin of expanded glass interrupted the capillary pore system and thus the transport processes, making the material more resistant (Figure 11). This would not be possible with other lightweight aggregates, because perlite and expanded clay have an open porosity for particles in corresponding sizes. This open porosity in turn leads to an increase water/fluid uptake, intensifying the corrosion processes. Anyhow, due to the low w/b ratio of UHPLC, the water/fluid uptake of the cement matrix was low, too. Thus, neither fluid could enter the microstructure and cause damage due to any chemical or physical attack (Figure 11). However, without the dense microstructure of UHPC, this type of microstructure would not be as resistant to frost and other chemical attacks with increasing volume due to the lack of expansion space.

## 6. Conclusions

The results of the durability tests show that UHPLC is excellently applicable as a structural material. The UHPLC can resist penetrating liquids and gases due to the dense cement paste structure. The lightweight aggregate did not affect the results of the material and the durability properties are comparable to a conventional UHPC (M3Q [1]).

- The compressive strength of the tested concretes was between 60 and 100 MPa with a bulk density of 1.6–1.9 kg/dm^3^. Thus, the performance was increased compared to conventional lightweight structural concretes. This resulted in a new class of materials that needs to be investigated in more detail.
- The analysis of the pore structure showed that the concretes investigated had increased porosity in the capillary pore (EG D1.6) and gel porosity (EG D1.8 and EG D2.0) regions. However, this was due to the porosity of the embedded expanded glass grains and not to the matrix. Therefore, the microstructure had few capillary pores.
- In frost tests, the high impermeability of the concrete structure ensured very good properties. Little weathering (68 g/m^2^) was measured
- The material showed very good resistance to sulfate attack at pH 3.5. However, other chemicals and pH ranges need to be investigated depending on the application.
- Abrasion by a grinding wheel, on the other hand, showed increased wear. The light aggregate, which had a low compressive strength, had a negative influence on the abrasion resistance. In the case of direct mechanical stress, it must therefore be checked in advance whether the resistance is sufficient.
- A rapid carbonation chamber was used to study the carbonation progress. A very low carbonation coefficient of 0.5 mm/year-1 was obtained. This allowed the concrete cover to be reduced.
- The chloride diffusion coefficient was also low and allowed structures to come into contact with salt water.

Resulting from these material characteristics applications are structural components in building construction and civil engineering as well as in interior and exterior applications. Furthermore, the material can be used in engineering structures such as bridges and high-rise buildings. However, it must be ensured that there is no direct abrasion stress, as the concrete is not sufficiently resistant in that case.

In order to consolidate the results of the investigations, other compositions of the UHPLC material must be investigated with regard to durability. In addition, data on thermal performance, like thermal conductivity or thermal diffusivity as well as hydrothermal performance [34], are needed. Another point is that the influence of the combination of UHPC matrix and lightweight aggregate on the fracture behavior and other mechanical properties, such as splitting tensile strength and fatigue tests, should be examined in regard to the influence of the combination of UHPC matrix and lightweight aggregate.

## Figures and Tables

**Table 1:** Mix design of UHPLC with different bulk density classes and UHPC [1].

Raw Materials	Unit	EG D1.6	EG D1.8	EG D2.0	UHPC [1]
Cement (CEM I 52.5 R)	(kg/m^3^)	771.90			775
Silica fume uncompacted	(kg/m^3^)	163.65			164
Superplasticizer	(kg/m^3^)	30.40 (3.9 % bwoc)			23.5 (3.0% bwoc)
Hydrophobing agent	(kg/m^3^)	15.45 (2.0 % bwoc)			-
Quartz flour	(kg/m^3^)	199.00			193
Quartz sand (0.125/0.5 mm)	(kg/m^3^)	-	338.99	627.76	946
Expanded glass (0.1/0.3 mm)	(kg/m^3^)	-	49.86	29.43	-
Expanded glass (0.25/0.5 mm)	(kg/m^3^)	122.45	128.83	67.61	-
Expanded glass (0.5/1 mm)	(kg/m^3^)	94.75	-	-	-
**Parameters of the Mix Design**					
Water/binder ratio	(kg/kg)	0.21			
Calculated fresh concrete density	(kg/m^3^)	1.56	1.86	2.07	2.31
Hardened concrete bulk density (20 °C; 65% r.h.)	(kg/m^3^)	1.59	1.79	1.97	2.30
Dry bulk density (oven-dry)	(kg/m^3^)	1.51	1.72	1.90	-

**Table 2:** Pore content of UHPLC and UHPC [3].

Type of Pores	EG D1.6	EG D1.8	EG D2.0	UHPC [3]
Gel pores (%) 0.001–0.03 µm	14.1	27.9	20.0	5.3
Capillary pores (%) 0.03 µm–10 µm	12.0	3.0	2.6	2.2
Air voids (%) 10 µm–100 µm	4.6	1.7	1.5	1.8
Total pore content (%)	30.7	32.6	24.1	9.3

## Data Availability

The data presented in this study are available on request from the corresponding author.
